# High-fidelity CNN-based surrogate modeling for wildfire spread forecasting

**DOI:** 10.1038/s41598-026-56080-w

**Published:** 2026-06-06

**Authors:** Saeed Nematshahi, Rui Fan, Amin Khodaei, Erin Belval, Ali Arabnya

**Affiliations:** 1 Department of Electrical and Computer Engineering, University of Denver, Denver, CO USA; 2 Rocky Mountain Research Station, U.S. Forest Service, Fort Collins, CO USA; 3 Quanta Technology, Raleigh, NC USA; 4 Sloan School of Management, Massachusetts Institute of Technology, Cambridge, MA USA

**Keywords:** Emergency risk assessment, Fire suppression, Power system resilience, Wildfire management, Wildfire spread forecasting, Engineering, Mathematics and computing, Natural hazards

## Abstract

The rising frequency of large-scale, destructive wildfires has significantly affected not only natural ecosystems but also critical infrastructure, human lives, and properties. Given the devastating and often irreversible environmental and financial consequences, researchers from diverse fields are actively seeking solutions to improve resilience against wildfires. Fire suppression, one of the most effective strategies to mitigate wildfire damage, relies heavily on rapid and high-fidelity forecasting of fire spread. These predictions are essential for planning evacuations by state or local emergency management agencies, implementing preemptive de-energization strategies for electric utilities, and coordinating fire containment efforts by firefighting teams. However, a significant bottleneck across all these planning processes is the significant computational burden imposed by high-resolution wildfire modeling, the demand for improved predictive accuracy, and the need to integrate diverse and large-scale datasets. Since response time is crucial for wildfire risk management, this paper proposes a deep learning-based surrogate model to predict fire spread in just a fraction of a second. We developed and trained a convolutional neural network (CNN) model that efficiently predicts wildfire propagation. The proposed model demonstrates high efficiency, achieving an F1 score of 0.92. The contributions of this paper are twofold: (1) a fast, high-resolution CNN model that can support wildfire-related public safety power shutoff (PSPS) planning for electric utilities, and (2) a practical tool for firefighting and evacuation teams to support rapid and data-informed risk assessment.

## Introduction

While wildfires have long been a natural ecological process, recent years have seen a marked escalation in their frequency, intensity, and spatial extent—driven primarily by climate change and the accelerating expansion of the Wildland-Urban Interface (WUI)^1^.

**Fig. 1:**
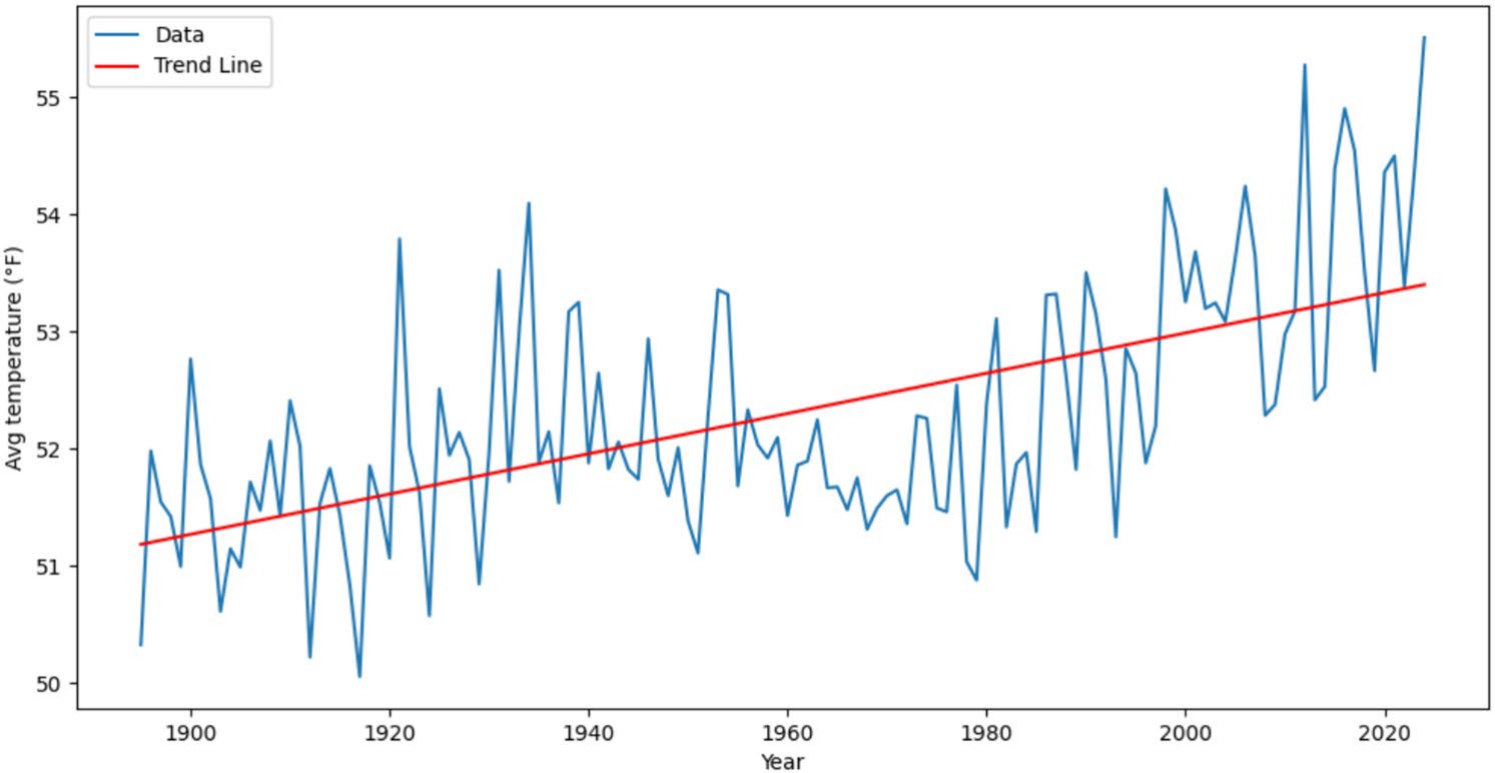
US average temperature of the year from 1985 to 2020.

According to data from the U.S. National Centers for Environmental Information, the average temperature in the United States during the twenty-first century has increased by approximately 1.64°F compared to the 20th-century baseline. Notably, all of the nine hottest years on record have occurred after 2006. Figure 1 illustrates the trend in the annual average temperature increase in the United States over the period of 1895–2024^2^. Elevated temperatures, coupled with low humidity and prolonged drought conditions, significantly increase the susceptibility of wildland areas to wildfire ignition and rapid spread.

**Fig. 2:**
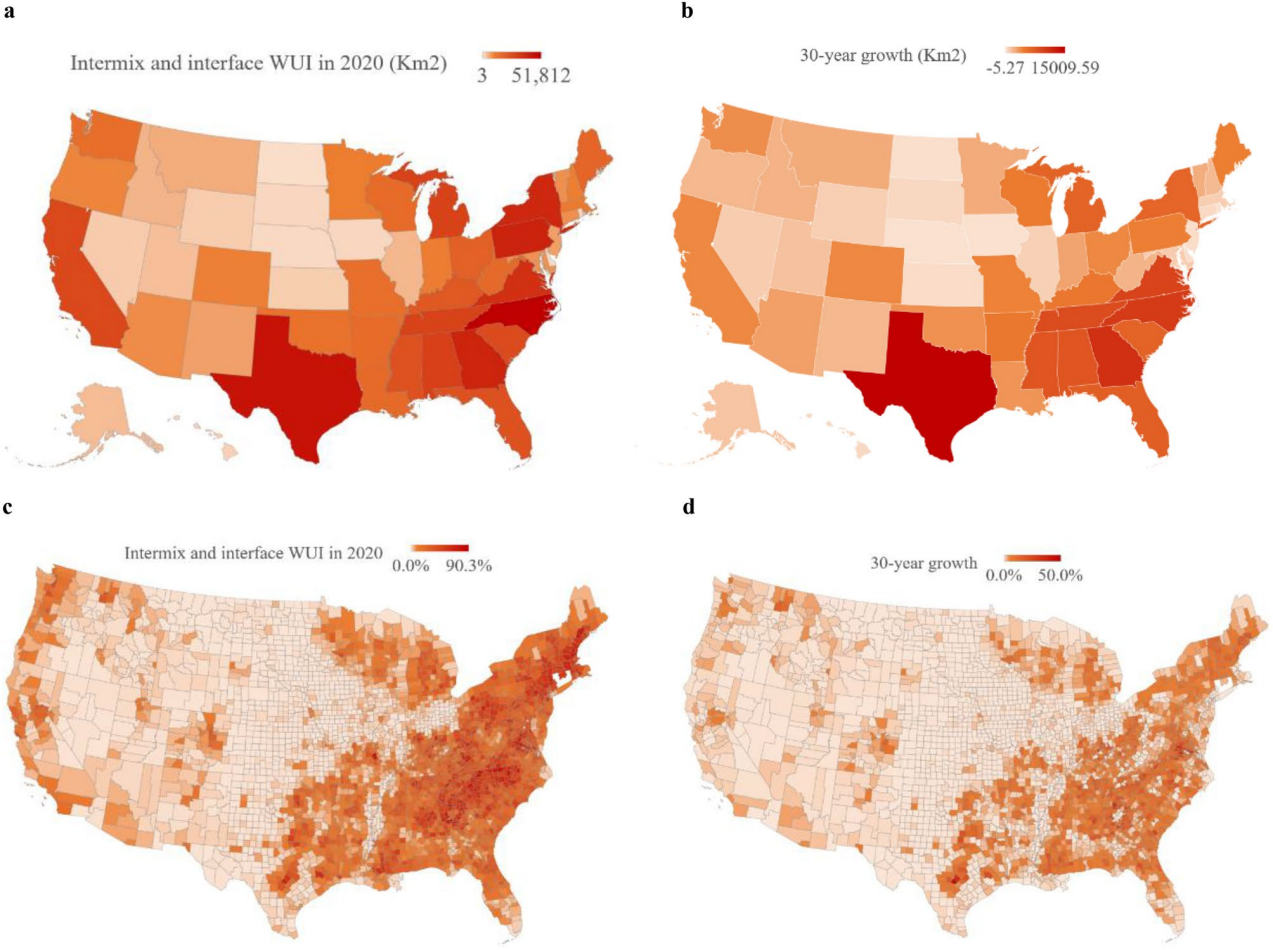
Wildland-Urban Interface distribution. (**a**) The 2020 WUI status in different states by km^2^. (**b**) The growth over the last thirty years in different states by km^2^. (**c**) The 2020 WUI status in different counties by percentage. (**d**) The growth over the last thirty years in different counties by percentage. *Note* The dataset did not include county-level data for Alaska and Hawaii.

Concurrently, the continued expansion of the WUI across the United States exacerbates the impacts of wildfires, including increased risk to human life^3^, property loss^4^, and damage to critical infrastructure^5–7^. Figure 2 presents the distribution of WUI areas by state and county within the United States^8^. The left figures depict the WUI status in 2020, while the right ones highlight the growth in WUI areas over the preceding 30 years. Overlaying the WUI map with the drought map identifies areas more susceptible to wildfire ignition and spread, highlighting regions at risk of costly wildfires.

Wildfire behavior modeling approaches are generally classified into two categories: physics-based fire dynamics simulators and data-driven models. Notable tools include IFTDSS (interagency fuels treatment decision support system), MTT (minimum travel time), FlamMap, FSim, FARSITE (fire area simulator), FireSim, FIRETEC, and WFDS (Wildland–Urban interface fire dynamics simulator). These models are widely used for wildfire behavior analysis, and each offers specific advantages. However, they require the integration of heterogeneous datasets, including landscape characteristics, fuel properties, and meteorological conditions. While this integration is feasible for long-term planning and risk assessment, it can pose significant challenges during emergency situations due to the time-intensive nature of data preparation and computationally intensive model execution. While conventional models remain important, their predictive performance can be limited without dynamic data updating and hybridization^9^. Authors in^10^ stated that future developments may involve machine learning algorithms to improve the wildfire models. Interpretable boosting models have achieved high accuracy in predicting wildfire spread scale while identifying key drivers such as humidity, wind speed, and suppression activity^11^. Deep learning frameworks have also enabled cross-scale forecasting of wildfire fronts with lead times up to 72 h^12^. In addition, specialized models such as TabNet outperformed general-purpose large language models in Fire Radiative Power prediction, highlighting the importance of domain-specific AI for operational wildfire forecasting^13^. In^14^, applied machine learning has been used to classify wildfire sizes in Siberia, with XGBoost achieving 88.8% accuracy and highlighting fuel dryness and urban proximity as key drivers. A recent study in^15^, developed a hybrid CNN–LSTM framework for short-term wildfire susceptibility forecasting, demonstrating improved predictive accuracy over conventional machine learning models by integrating meteorological and geospatial drivers.

The authors in^16^ introduced the Next Day Wildfire Spread dataset—a publicly available resource encompassing thousands of wildfire events across the contiguous United States from 2012 to 2020. This dataset is designed for tasks such as statistical analysis for land management and disaster preparedness. It captures the spatial progression of wildfires by providing daily snapshots, enabling comparative analysis of fire spread over two consecutive days. Building on this resource^17^, employed the dataset to develop the Attention Swin U-Net with Focal Modulation (ASUFM) for next-day wildfire spread prediction. Similarly,^18^ applied three machine learning algorithms—decision tree regressor, XGBoost regressor, and artificial neural network (ANN)—to model fire spread in the next day, while^19^ introduced an explainable convolutional neural network (CNN) architecture with atrous spatial pyramid pooling (CNN-ASPP) for the same purpose. While these studies demonstrate promising results, they are constrained by the historical dataset’s inherent limitations—namely, a prediction horizon restricted to a single day and a spatial resolution of 1 km. In^20^, the authors proposed a deep convolutional inverse graphics network (DCIGN) for modeling wildland fire spread at a spatial resolution of 1 km. The model requires approximately 18 to 51 h for training and takes around 185 s to generate a prediction for a wildfire scenario. It achieves an F1 score of 0.78 but is limited to forecasting fire spread over a two-day horizon. In contrast, real-world wildfires often span multiple days to be fully contained, necessitating faster, more accurate, and higher-resolution predictive capabilities to inform tactical decision-making by emergency response teams across multiple sectors. Authors in^21^ developed a neural network framework based on the MA-Net architecture for three-day-ahead prediction, reporting an F1 score of 0.67. Large benchmark datasets integrating remote sensing imagery, weather, topography, land cover, and fuel information now support tasks such as active fire detection, burned area mapping, and next-day progression prediction^22^. To the best of our knowledge, no existing study has yet presented a machine learning framework that can accurately predict large-scale wildfire spread while remaining fully independent of data sources that are often costly, delayed, or operationally restrictive. Most current wildfire prediction models rely heavily on one or more of three external inputs: unmanned aerial vehicles (UAVs) for localized real-time observations, historical wildfire databases for training on past fire behavior, or frequently updated satellite imagery for continuous monitoring of active events. Although these inputs can improve forecasting performance, they also introduce important limitations related to coverage, scalability, response latency, weather interference, data availability, and deployment cost. UAV-based systems are typically constrained to small operational areas and require field coordination, while satellite products may suffer from revisit delays, cloud obstruction, smoke contamination, or insufficient spatial resolution during fast-moving events. Similarly, historical wildfire datasets may not generalize well to unprecedented fire behavior driven by changing climate conditions, novel fuel patterns, or region-specific topography. Therefore, a robust machine learning approach that can forecast large-scale wildfire spread using readily available static or low-frequency environmental data would represent a significant advancement for rapid-response operations and geographically transferable wildfire management.

In this work, we present a CNN-powered surrogate model that achieves an F1 score of 0.92, operating at a spatial resolution of 120 m with a prediction time of fractions of a second. The model is adaptable to different geographic regions and was fully trained in under 4 h on a standard personal computer equipped with a 13th Gen Intel® Core™ i7-13700 CPU (2.10 GHz) and 16 GB of RAM, without the need for GPU acceleration. The model was trained on a dataset comprising 1,217 wildfire scenarios and demonstrates robust performance across both small- and large-scale wildfires, with no observable degradation in predictive accuracy based on fire size. Moreover, it effectively captures a wide variety of wildfire shapes and spatial configurations, highlighting its generalizability and spatial adaptability. This model offers valuable contributions to wildfire emergency risk assessment and mitigation strategies. It can support Public Safety Power Shutoff (PSPS) planning by enabling electric utilities to proactively de-energize power lines in areas projected to be affected by wildfire spread. Additionally, it aids in the development of evacuation plans, particularly when wildfires are forecasted to encroach upon WUI zones within the coming hours or days. Most critically, it provides firefighters with timely and high-resolution predictions of fire progression, facilitating more informed and effective fire suppression planning and resource deployment.

## Results

### Model establishment

**Fig. 3:**
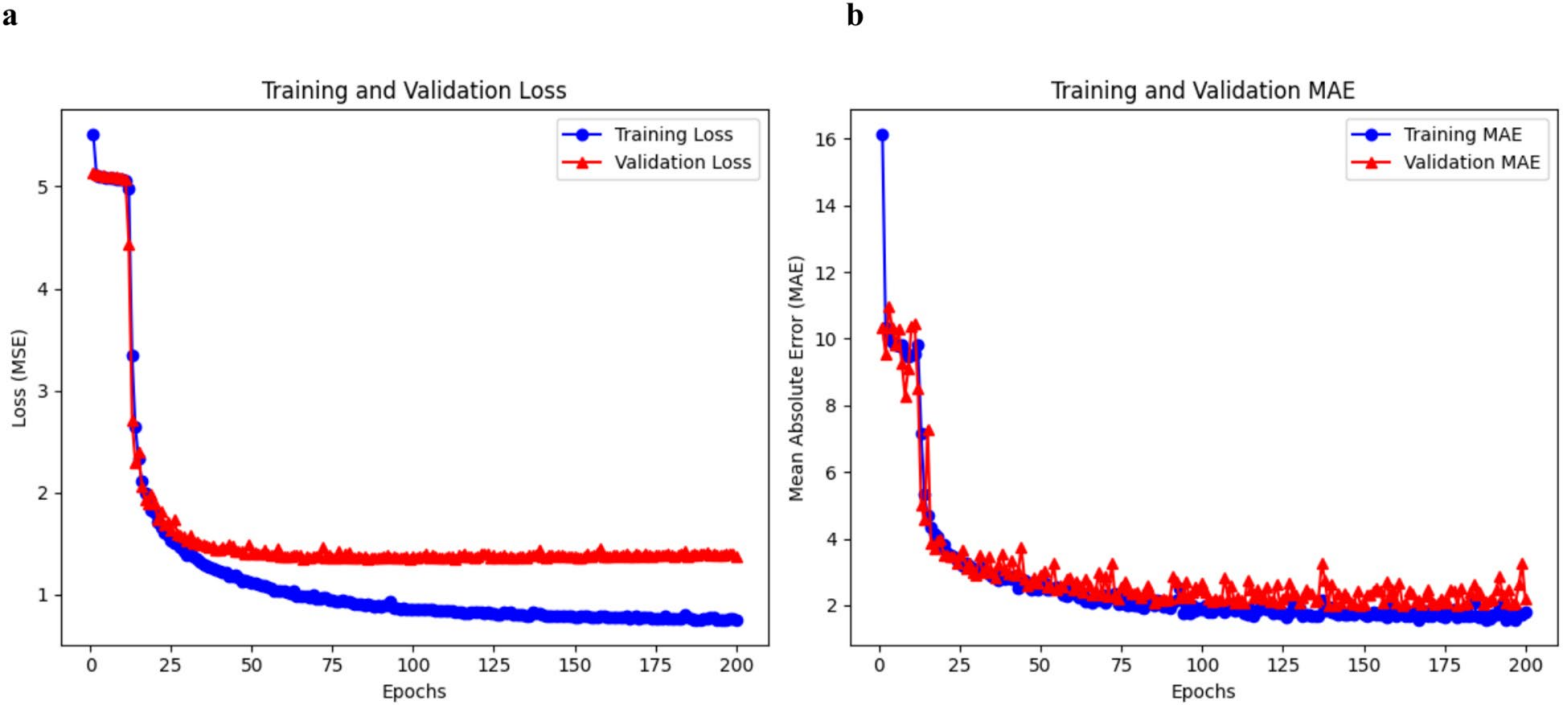
Model MSE and MAE through the epochs. (**a**) Training and validation MSE loss. (**b**) Training and validation MAE.

The model begins by extracting features most relevant to wildfire propagation, learning their spatial and contextual influence on fire spread across 1,217 simulated scenarios. During training, it aims to minimize the loss function, which is computed over the entire prediction domain of 464 × 516 pixels (239,424 total pixels). Figure 3 illustrates the training and validation mean squared error (MSE) across 200 epochs, demonstrating progressive improvement in model accuracy. Notably, the validation curve indicates that acceptable prediction performance can be achieved even after approximately 100 epochs, suggesting efficient convergence. The output of the model would be a continuous variable for every single pixel, indicating the probability of fire occurrence. A subsequent post-processing step thresholds the continuous model output using a cutoff value of 0.5, where pixels with values greater than 0.5 are classified as burned and those below are considered unburned, thereby generating a binary fire spread map. The threshold value was selected based on empirical evaluation across thousands of wildfire scenarios by maximizing the overall F1 score. Since the network output represents normalized fire occurrence likelihood values between 0 and 1, the selected threshold also provides a balanced and interpretable decision boundary between burned and unburned classes.

**Fig. 4:**
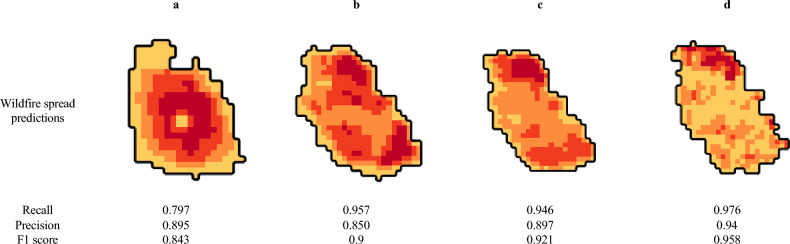
Efficiency progress during epochs. (**a**) Prediction of burned area after 15 epochs. (**b**) Prediction of burned area after 30 epochs. (**c**) Prediction of burned area after 45 epochs. (**d**) Prediction of burned area after 200 epochs. *Note*: The different colors represent the regression output and do not correspond to the temporal progression of the fire. All pixels within the figure indicate burned areas. As training progresses, the model produces increasingly sharper burn perimeters, reflecting improved accuracy.

To provide a clearer illustration of the model’s performance progression, Fig. 4 visualizes the predicted wildfire spread at selected training epochs—15, 30, 45, and 200. During the training process, the number of true positives exhibits an increasing trend from 1181 at epoch 15 to 1444 at epoch 200, while the number of false negatives shows a decreasing trend from 299 to 36 over the same period. The results demonstrate a progressive refinement in prediction accuracy, with the F1 score improving from 0.843 at epoch 15 to 0.958 at epoch 200. This highlights the model’s ability to achieve reasonably accurate predictions at early training stages, offering significant advantages for time-sensitive applications such as real-time risk assessment. For instance, the model can be trained from scratch using up-to-date weather data, and after only 15 epochs (approximately 15 min of training time), it can provide a practical forecast of fire spread. When computational resources and time permit, training can be extended up to 200 epochs to reach maximum performance. While the model has also been trained for 500 epochs, results indicate diminishing returns, with no significant improvements in predictive performance beyond 200 epochs. In addition, for a long-term wildfire mitigation plan, it can be executed through different seasons with different weather streams to receive a thorough wildfire risk analysis in the area of study^23^.

### Performance

Our surrogate model is a fast-response yet accurate wildfire spread predictor. To evaluate its generalization performance, we have selected five random instances to depict the accuracy of our approach. The five wildfire scenarios presented in Table 1 were randomly selected from the held-out validation subset following model training to demonstrate the predictive performance of the proposed framework across different wildfire sizes and spatial propagation patterns. Each scenario was initially simulated using FARSITE, and the corresponding fire spread was then predicted by our CNN model. Table 1 presents side-by-side visualizations of the ground-truth FARSITE outputs and the CNN-generated predictions, allowing for direct visual and spatial comparison. The size of the wildfire spread for different scenarios is diverse, so we present the spread shapes in the same size, but the actual size is mentioned beneath the burned area. The smallest case is 2,775 acres, while the largest case among the five selected scenarios is 12,188 acres. The model’s accuracy in predicting the wildfire spread is not dependent on the wildfire size or even shape, which shows its capability to handle both small and large-scale wildfires without losing accuracy. Notably, Ignition 23 on line 3 includes an internal unburned pocket (a “hole”) within the fire perimeter—typically a challenging feature to predict. Nevertheless, the model successfully identifies this complex pattern, further illustrating its capability to capture nuanced fire behaviors.

**Table 1:** Wildfire spread predictor analysis.

Instance	Burned area		Efficiency metrics		
	Actual	Prediction	Recall	Precision	F1 score
Ignition 3 on line 41			0.976	0.94	0.958
Ignition 27 on line 10			0.940	0.931	0.936
Ignition 23 on line 3			0.965	0.872	0.916
Ignition 17 on line 18			0.972	0.966	0.969
Ignition 6 on line 40			0.884	0.978	0.929

This entire area encompasses approximately 842,000 acres, bounded by latitudes 37.6° to 38.1° and longitudes − 120.7° to − 120.0°

Next to every instance, the confusion matrix and efficiency matrix are mentioned. Since the number of true negatives (TNs) is very high compared to other values in the confusion matrix, the accuracy for the scenarios always remains over 99%, rendering it an uninformative metric in this context. Instead, recall and F1 score are particularly useful metrics when evaluating the efficiency of wildfire spread prediction models, especially in scenarios where the dataset is imbalanced and the consequences of missing a fire-affected area are severe. Recall is crucial when the priority is to detect all areas that the wildfire might reach, minimizing false negatives, which is important for emergency response and evacuation planning. However, focusing solely on recall can lead to many false positives. The F1 score addresses this by providing a balanced metric that combines both precision and recall, making it valuable when both false alarms and missed detections carry significant consequences. These metrics are especially relevant in grid-based or spatiotemporal prediction tasks where each unit (e.g., pixel or zone) is classified as fire-affected or unaffected over time.

Across all scenarios, the model achieved an average Recall of 0.925 and an average F1 score of 0.92, demonstrating high reliability in identifying burned areas at a spatial resolution of 120 m.

## Discussion

Our study aims to predict wildfire spread in a fast yet accurate manner. We developed a CNN-powered surrogate model that estimates the burned area upon receiving the ignition point. Wildfire behavior modeling typically requires the integration of diverse datasets—such as topography, vegetation, and weather—and involves complex physical formulations, making the process computationally intensive and time-consuming. In emergency response scenarios, however, rapid and precise fire spread predictions are critical for effective decision-making. While traditional physics-based models embedded in wildfire simulation tools provide accuracy, the data preparation and simulation process often take several hours. In contrast, the proposed surrogate model delivers wildfire spread predictions in a fraction of a second, achieving high performance with an average F1 score of 0.92 and a Recall of 0.93, making it a practical and scalable tool for real-time emergency planning.

The predictive high fidelity and rapid inference capability of the proposed model make it particularly well-suited for real-time applications in emergency response planning during wildfire events. Its utility extends across multiple facets of wildfire resilience, supporting proactive decision-making for fire suppression strategies, evacuation planning, and infrastructure protection.

First, in the context of fire suppression strategy development, the model facilitates anticipatory planning for strategic coordination of grounded and aerial suppression resources (e.g., water and retardant drops). Also, in case of a large-scale wildfire, they can immediately ask for resources across municipal, state, and federal entities.

Second, it can improve evacuation planning by highlighting areas that are likely to be affected and helping determine safe and efficient evacuation routes. This includes issuing timely alerts, managing traffic flow, and ensuring that shelters are prepared to receive displaced people, animals, and vulnerable populations.

Third, the model can inform power system operational decisions, such as implementing public safety power shutoffs (PSPS) or dynamically adjusting line ratings based on fire intensity profiles beneath the power lines^24^. Such decisions are vital to minimizing ignition risks from electrical infrastructure and preserving grid stability during evolving wildfire threats.

Fourth, road access control and traffic management are also enhanced through the model’s predictive analytics. By identifying intra-zoon road networks and potential choke points, emergency planners can implement preemptive road closures and diversions to both expedite evacuation and facilitate unimpeded access for emergency responders.

Collectively, the model’s performance characteristics align with the operational needs of contemporary wildfire response systems, underscoring its potential to be embedded within decision-support tools for resilience planning under wildfire hazard scenarios.

The predicted binary wildfire spread map is intended to function as a foundational spatial hazard layer within broader operational decision-support systems rather than as a standalone autonomous decision-making tool. For PSPS applications, the predicted fire perimeter can be spatially integrated with utility infrastructure maps, transmission corridors, load criticality information, and operational reliability constraints to support risk-informed de-energization strategies. Similarly, for evacuation planning, the forecasted wildfire spread can be combined with transportation networks, population distribution, shelter availability, and traffic management models to identify vulnerable communities, estimate evacuation bottlenecks, and dynamically support route planning. Therefore, the proposed framework provides the rapid wildfire hazard forecasting component upon which higher-level operational and optimization models can subsequently operate.

The reported Recall and F1 score values quantify the agreement between the proposed CNN surrogate model and the FARSITE-generated wildfire simulations used as reference data. Therefore, these metrics should be interpreted as indicators of surrogate-model fidelity relative to the underlying physics-based simulator rather than direct validation against observational wildfire spread data.

A limitation of the proposed framework is that the CNN surrogate model is trained on wildfire simulations generated by the FARSITE physics-based simulator. Consequently, the model inherently learns and reproduces the assumptions, simplifications, and potential biases embedded within the underlying simulator. Although FARSITE is a widely validated wildfire modeling tool, real-world wildfire behavior may involve additional complexities—such as abrupt atmospheric changes, ember spotting, plume-dominated fire dynamics, suppression interventions, and fine-scale fuel heterogeneity—that are not fully represented in the training data. For example, it relies on semi-empirical correlations of fire behavior and is thus incapable of predicting extreme wildfire behavior, such as fire whirls, fire merging, and rapid fire spread on steep slopes^25–27^. Nevertheless, the proposed deep learning framework is fully adaptable and can be trained using alternative wildfire datasets or operational fire observations as such datasets become available. In this work, FARSITE-generated wildfire scenarios were employed because publicly available datasets containing large numbers of high-resolution and temporally indexed wildfire spread samples suitable for deep learning training remain extremely limited.

## Methods

### Dataset

To develop and train our CNN-powered wildfire spread prediction model, we constructed a diverse and high-resolution wildfire simulation dataset. The dataset was generated using FARSITE (Fire Area Simulator)—a widely adopted wildfire behavior model developed by the U.S. Forest Service. It integrates topography, fuel characteristics, and weather conditions to simulate fire spread and produces two-dimensional, GIS-compatible outputs for fire management and planning applications.

Our dataset comprises 1,217 wildfire scenarios covering approximately 842,000 acres in central California, specifically between latitudes 37.6° N to 38.1° N and longitudes − 120.7° W to − 120.0° W. This region includes parts of the Sierra Nevada foothills and the San Joaquin Valley, encompassing a diverse landscape with oak woodlands, grasslands, and coniferous forests—offering a wide range of fuel types for modeling fire behavior. The area’s Mediterranean climate, characterized by hot, dry summers and wet winters, along with strong seasonal winds and prolonged drought conditions, makes it especially susceptible to wildfires. The proximity to rural communities, agricultural zones, and Yosemite National Park further underscores its importance as a case study for wildfire risk assessment and mitigation planning.

We overlaid the IEEE 30-bus transmission network onto the designated study region and introduced random ignition points along the transmission lines, under the assumption that each wildfire event would be fully contained within 96 h^28^. Each ignition point represents a potential grid-ignited wildfire event. The geographic distribution of transmission infrastructure across diverse terrain within the study area ensures that the dataset captures a wide range of fire spread dynamics, enhancing the generalizability and adaptability of the trained model to different regions within the study zone.

### CNN model

**Fig. 5:**
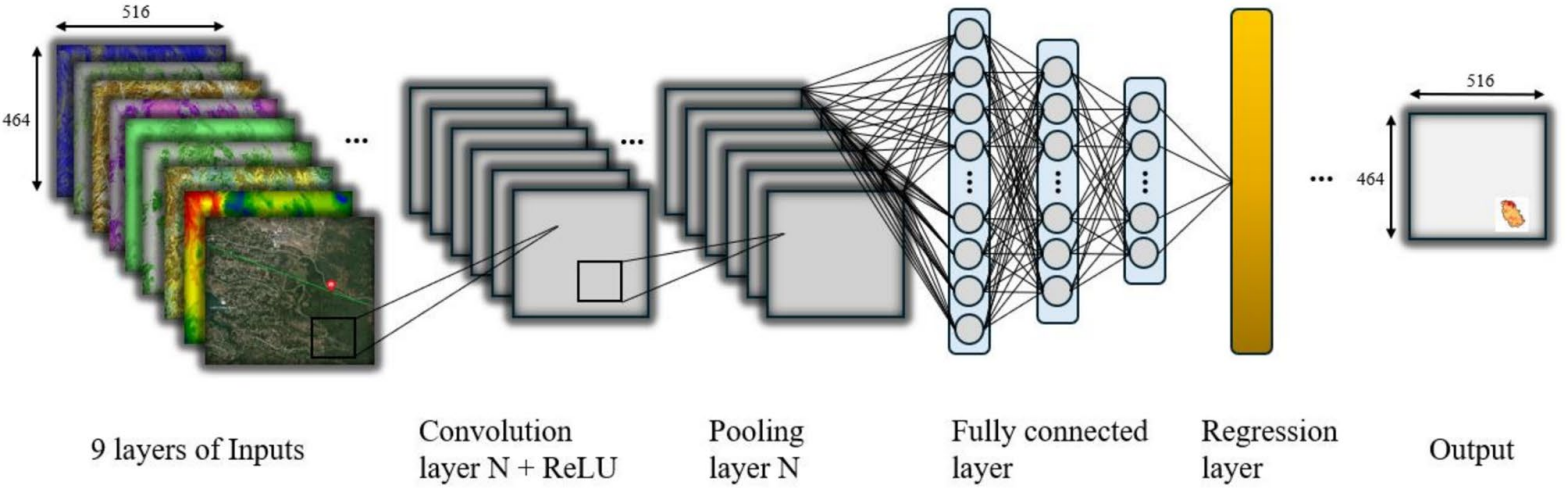
The architecture of convolutional neural networks (CNNs). Two convolutional layers and two pooling layers are adopted in the wildfire spread model.

The proposed convolutional neural network (CNN) is architected to predict wildfire spread, with a particular focus on crown fire activity, which reflects the intensity and spatial extent of fire propagation through forest canopies. Figure 5 depicts the architecture of the proposed CNN, indicating the input layers, convolutional layers, pooling layers, fully connected layers, regression layer, and the output.

The model ingests two distinct categories of input data: fixed input layers and variable input layers.

The eight fixed geospatial layers are derived from the LANDFIRE database. These layers represent the standard landscape (LCP) variables required for high-fidelity wildfire behavior modeling: elevation, slope, aspect, fuel model, canopy cover, canopy height, crown base height, and crown bulk density. These layers collectively provide the static environmental context for the 842,000-acre study area in central California. To ensure consistent feature weighting and numerical stability during the training of the CNN, each geospatial layer was normalized using Min–Max scaling to a range of [0, 1]. For the concatenation process, these eight normalized geospatial layers were stacked channel-wise alongside the one-hot encoded ignition map, which indicates the spatial origin of the fire. This resulted in a composite input tensor of dimensions 464*516*9* for each simulation instance. While physics-based simulators (such as FARSITE) require the integration and preprocessing of heterogeneous datasets—including landscape, fuel, and dynamic meteorological conditions—for every simulation run, our surrogate model performs this integration only once. By utilizing these layers as a fixed environmental backbone, the model requires only a new ignition map to perform inference, allowing it to generate high-resolution forecasts (120-m resolution) in fractions of a second.

**Fig. 6:**
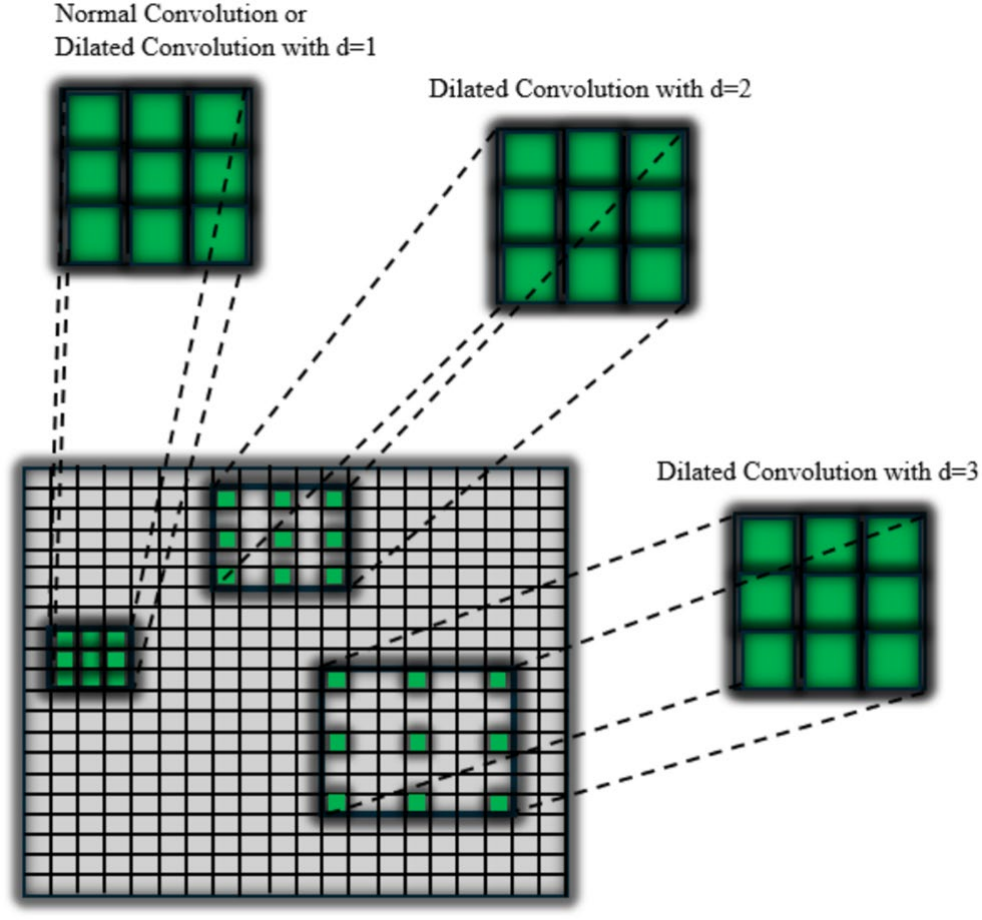
The dilated convolutional architecture. A higher dilated convolution reflects more information from the surrounding pixels, which makes the CNN model sharper yet more reliable.

The model architecture is specifically designed to capture both fine-grained and broad-scale spatial patterns. Following the input concatenation, the network utilizes a sequence of dilated convolutional layers with a dilation rate of 10 and ReLU activation functions, beginning with 32 filters and increasing to 64 filters. Figure 6 shows how dilated convolution captures the information from the surrounding pixels. This use of dilation expands the receptive field, allowing the model to incorporate long-range spatial dependencies (e.g., terrain-driven fire spread) without a proportional increase in computational cost. Each convolutional block is followed by max-pooling layers, which reduce the spatial dimensions (e.g., from 464 × 516 to 232 × 258, and then to 116 × 129), effectively extracting dominant features such as fuel continuity and slope gradients.

To reconstruct high-resolution predictions, the architecture employs transposed convolutional layers (Conv2DTranspose) with 64 and 32 filters, along with upsampling layers, to progressively restore the spatial dimensions to the original input size. A final 1 × 1 convolutional layer with linear activation produces a single-channel output representing the predicted crown fire activity as a continuous variable across the spatial grid.

The model is compiled using the Adam optimizer with a learning rate of 0.0005, and trained with mean squared error (MSE) as the primary loss function. Mean absolute error (MAE) is employed as an additional performance metric to assess prediction accuracy during both training and validation phases.

### Training and prediction process

The model is trained on a dataset architecture in which fixed environmental inputs—such as topography, fuel characteristics, and weather conditions—are replicated across all instances, while variable ignition points and their corresponding crown fire outputs are loaded from separate directories. Each pair constitutes a single training instance. To promote model generalization, prior to training, the 1217 wildfire scenarios were randomly shuffled, and 20% of the dataset was reserved as a validation subset. The reported performance metrics, including the average Recall and F1 score, were computed on this held-out validation dataset, which was excluded from the model parameter update process during training. The model is trained over 200 epochs with a batch size of 8, using a 20% validation split. This configuration enables the model to effectively learn how ignition locations interact with static geospatial features to influence wildfire spread dynamics.

During training, model performance is evaluated on the held-out validation set using mean squared error (MSE) and mean absolute error (MAE) as loss metrics. In this case, the loss function per pixel is:

1 $$L = \frac{1}{N}\mathop \sum \limits_{i = 1}^{N} \left( {y_{i} - \hat{y}_{i} } \right)^{2}$$

where ${y}_{i}$ is the ground truth value for pixel i, ${\widehat{y}}_{i}$ is the predicted value from the network output, and N is 464 × 516 pixels per sample × number of samples in batch.

Once trained, the model generates spatial crown fire prediction maps, accurately estimating the progression of wildfire from given ignition points under specific environmental conditions. The final trained model is saved for future inference tasks, enabling real-time wildfire forecasting that supports emergency operations such as fire suppression planning, evacuation routing, and public safety power shutoff (PSPS) decision-making.

### Efficiency metrics

The study area comprises 239,424 pixels, with output for each pixel labeled as 1 to indicate burned area and 0 for unburned. For each wildfire scenario, a confusion matrix was generated by comparing the model’s predicted burn map to the ground truth data from the simulation. Due to the overwhelming prevalence of TNs—reflecting the relatively small proportion of burned pixels relative to the total area—the overall accuracy consistently exceeds 0.99, making it an insufficient standalone metric for model evaluation in this context. To more effectively assess model performance, we focused on Recall and the F1 score as primary efficiency metrics.

2 $$Recall = \frac{True\,Positives}{{True\,Positives + False\,Nagatives}}$$

3 $$F1 \,score = 2 \cdot \frac{Precision \times Recall }{{Precision + Recall}}$$

where,

4 $$Precision = \frac{True\,Positives}{{True\,Positives + False\,Positives}}$$

## Data Availability

The landscape data is retrieved from LandFire, and the weather stream is accessible from the National Solar Radiation Database (NSRDB) at https://nsrdb.nrel.gov/ with hourly intervals. The dataset of 1217 wildfire simulations is available at Zenodo at https://zenodo.org/records/15707374^29^.

## Code availability

The developed codes are available in the following repository. https://zenodo.org/records/15707691^30^.
